# Early induction of C/EBPβ expression as a potential marker of steroid responsive colitis

**DOI:** 10.1038/s41598-019-48251-9

**Published:** 2019-09-11

**Authors:** Mushref Bakri Assas, Scott Levison, Joanne L. Pennock

**Affiliations:** 1 Faculty of Applied Medical Sciences, Department of Medical laboratory technology, King AbdulAziz University, Jeddah, Saudi Arabia; 2 School of Biological Sciences, Faculty of Biology Medicine and Health, University of Manchester, Manchester, UK; 3 Manchester Royal Infirmary, Manchester University NHS Foundation Trust, Manchester, UK; 4 Lydia Becker Institute of Immunology and Inflammation, School of Biological Sciences, Faculty of Biology Medicine and Health, University of Manchester, Manchester, UK

**Keywords:** Drug development, Immunology, Inflammation

## Abstract

The precise mechanism of hydrocortisone immune regulation in the management of colitis is poorly understood. Whilst not without limitations, its ability to suppress pathology and rapidly improve patient clinical outcome is key. We were interested in identifying early markers of therapeutic responsiveness in order to identify patients’ refractory to therapy. Chronic Th1-driven colitis was induced in AKR/J mice using a parasite infection, *Trichuris muris*. 35 days post infection, mice were treated with low dose hydrocortisone (2 mg/kg/) i.p. on alternate days. Response to therapy was assessed at a systemic and tissue level day 45 post infection. Histopathology, gene and protein analysis was conducted to determine cytokine and transcriptional profiles. The colonic transcriptional profile in steroid treated mice showed significant upregulation of a small subset of T cell associated genes, in particular C/EBPβ, CD4, IL7R and STAT5a. Despite no change in either transcription or protein production in downstream cytokines IFN γ, TNFα IL-17 and IL-10, hydrocortisone treatment significantly reduced colonic pathology and restored colonic length to naïve levels. As expected, steroid treatment of chronic gut inflammation generated significant immunosuppressive effects characterized by histological improvement. Low dose hydrocortisone induced significant upregulation of a subset of genes associated with T cell maintenance and regulation, including C/EBPβ. These data suggest that enhanced expression of C/EBPβ may be one of a subset of early markers demonstrating an immune regulatory response to hydrocortisone therapy, potentially by stabilization of Treg function. These observations contribute to our understanding of the immune landscape after steroid therapy, providing a potential markers of therapeutic responders and those refractory to hydrocortisone treatment.

## Introduction

Administration of corticosteroids is an established first line treatment for many inflammatory diseases, although their precise mechanism of action is not clear and efficacy can be variable and unpredictable. Transmural intestinal inflammation results from innate and adaptive immune cell infiltration with accumulation of pro-inflammatory cytokines^1^. The interaction of effector CD4^+^ T-helper cells (e.g. T_H_1 and T_H_17), neutrophils and macrophages can disrupt intestinal homeostasis and epithelial integrity, to precipitate and perpetuate inflammation^2–7^.

In an advancing era of personalized medicine, the ideal scenario is to fully understand the mechanism of action of each therapeutic approach in order to match a treatment to the biological pattern of an individual’s disease. Approximately 20% of IBD patients are refractory to corticosteroid therapy, and although genetic factors have been identified relating to therapeutic responses^8,9^ there are still no identified biomarkers to monitor therapy or predict responses. It is known that macrophages, neutrophils and lymphocytes all play a role in steroid resistance, with granulocyte/monocyte adsorptive apheresis a viable option for non-pharmacological intervention^10^ in these patients.

By better understanding local protein and gene responses in the inflamed gut after steroid treatment, key elements may be identified as potential markers of unresponsiveness. Additionally, a better understanding of the regulatory mechanisms which enhance mucosal healing could lead to new therapeutics and help overcome therapeutic failure.

In this study we investigated how the colonic immune phenotype changes in response to corticosteroid therapy during colitis. We chose to study the effect of corticosteroid treatment in a mouse model of chronic colitis^11^. Given that NICE guidelines recommend steroid treatment to induce remission in patients on first presentation or having suffered one exacerbation in the last twelve months, we chose this mild-to-moderate model over the more commonly used DSS induced colitis, as the infection induces a low number of T regulatory cells and the epithelium remains intact. The key parameters studied were immune gene expression, cytokine protein expression, pathology and overall clinical outcome.

## Results and Discussion

### Therapeutic intervention with hydrocortisone improved the colonic and systemic phenotype of chronic *T*. *muris*-induced colitis

**Figure 1:**
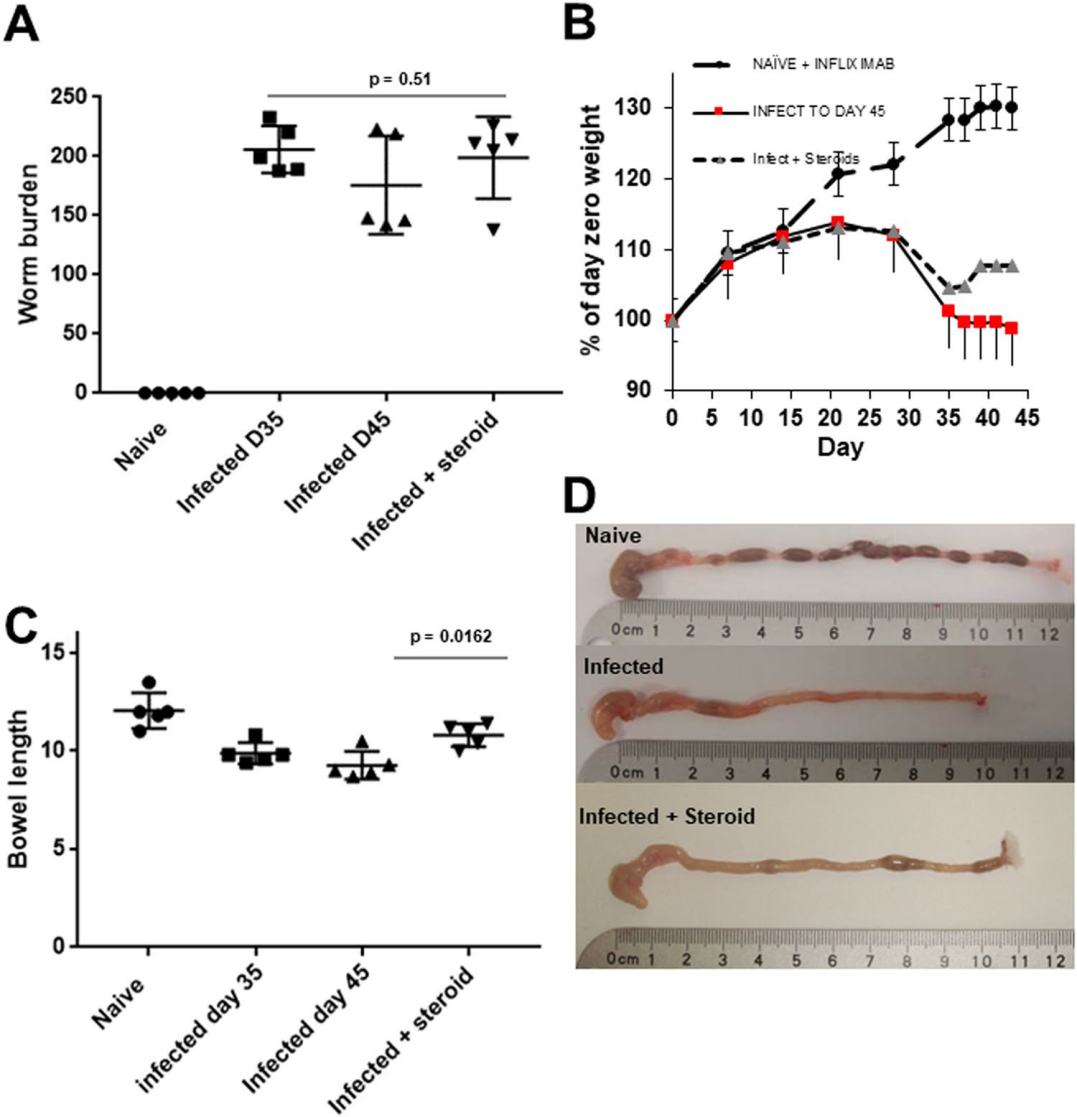
Steroid intervention improved colonic phenotype and body weight of AKR/J mice infected with *T*. *muris*. AKR/J were infected with 300 *T*. *muris* ova, and studied to 45 days post-infection. Steroids were administered from day 35 p.i. on alternate days. (**A**) The worm burden was not significantly different across infected groups (ANOVA p = 0.51). (**B**) Both infected groups displayed significant weight loss compared to naïve AKR prior to the initiation of treatment on day 35 p.i. (ANOVA, p < 0.0001). Steroid treatment initiated significant weight loss recovery. (**C**) Colonic shortening observed post-infection was ameliorated by steroid treatment. (**D**) Macroscopic colonic appearances across experimental cohorts demonstrated biological differences (shortened, more oedematous, lack of ingesta) in untreated and *T*. *muris* infected AKR. The data are expressed as the mean ± SD. n = 5 per group. Data representative of two independent experiments.

Hydrocortisone was administered from day 35 post-infection with *T*. *muris*, when chronic inflammation was established. Persistent worm burden was seen in all infected AKR/J (193 +/− 28, Fig. 1A); immune suppression did not induce worm expulsion. Prior to treatment, no significant weight difference between infected cohorts was observed although as expected infected cohorts displayed significant weight loss compared to naïve mice (ANOVA, p < 0.0001, Fig. 1B). Steroid treated mice showed some weight loss recovery during treatment although this was not significant (Fig. 1B).

**Figure 2:**
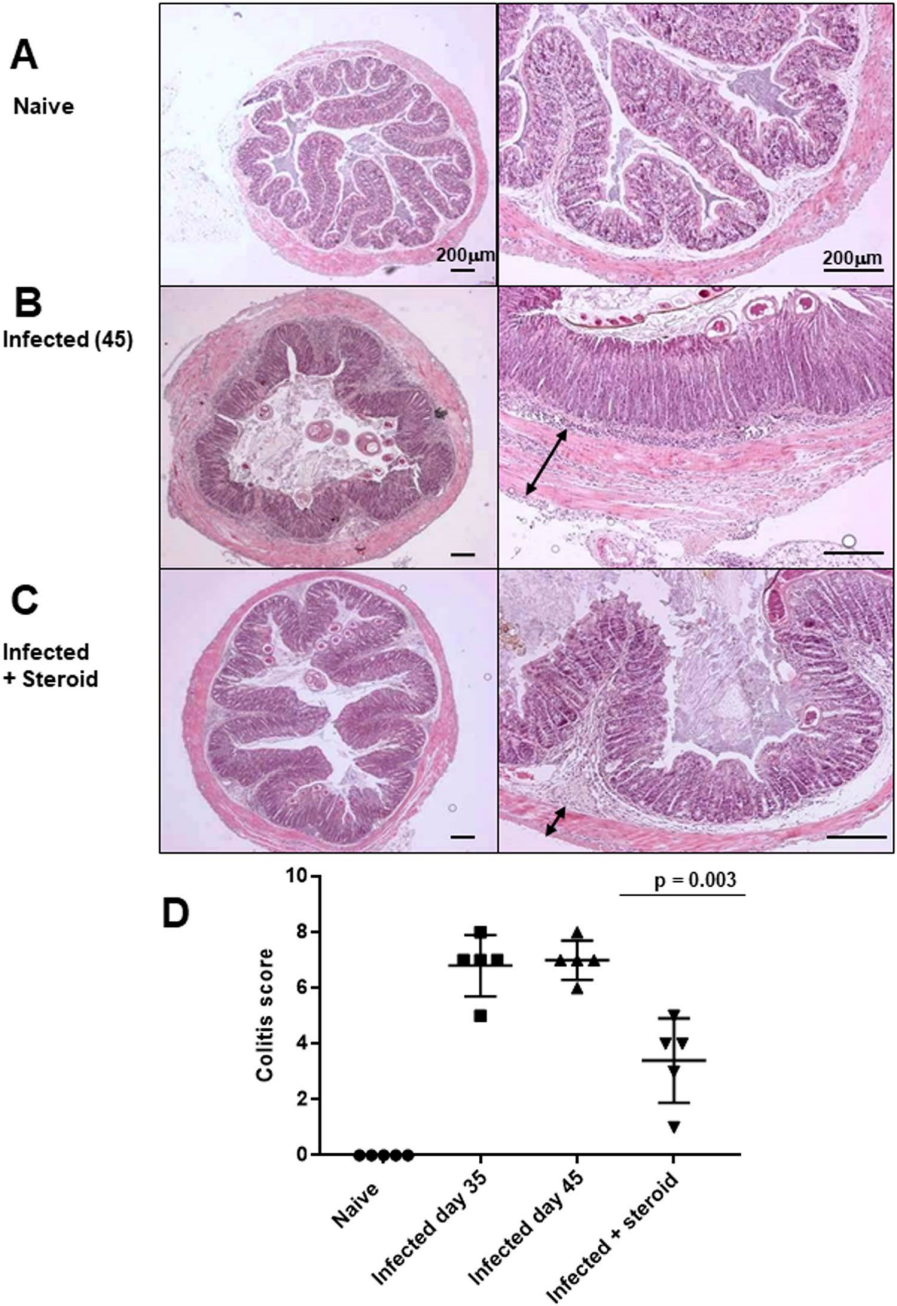
Hydrocortisone intervention significantly improves histological colitis score in *T*. *muris* infected mice at day 45 p.i. (H&E, scale bar = 200 μm). (**A**) Normal colonic tissue in naïve mice. (**B,C**) Luminal and mucosal helminths and transmural colonic inflammatory changes were seen post *T*. *muris* infection in AKR/J mice. A reduction of muscle layer oedema, (double headed arrows) accompanied by a reduction in submucosal oedema and crypt hyperplasia was identified following steroid treatment of infected mice (**C**). (**D**) The histological assessment of mucosal architecture, ulceration, crypt abscesses, goblet cell depletion, cellular infiltration and tissue oedema was quantified. A significant reduction in colitis severity was seen following steroid treatment. Data represented as mean +/− SD, representative of two independent experiments, n = 5 per group.

Macroscopic colonic appearances between groups demonstrated clear differences. As expected, altered stool pellets, gross inflammatory changes (oedema, colonic tissue texture), and colonic shortening were seen in untreated infected mice (Fig. 1C,D). Colonic shortening at day 45 was partially resolved by steroid treatment (p = 0.006). Histologically, mild-to-moderate inflammatory changes were observed in all infected groups included transmural tissue oedema and leukocytic infiltration (lymphocytes, macrophages, neutrophils), prominent mucosal and submucosal reactive lymphoid aggregates, and colonic crypt hyperplasia and hypertrophy (Fig. 2). Steroid treated AKR/J mice showed histological improvement (p = 0.0047) due to less oedema, reduced cell infiltration, and maintenance of goblet cell numbers (data not shown).

### Hydrocortisone treatment significantly upregulated antigen specific IL2 production

**Figure 3:**
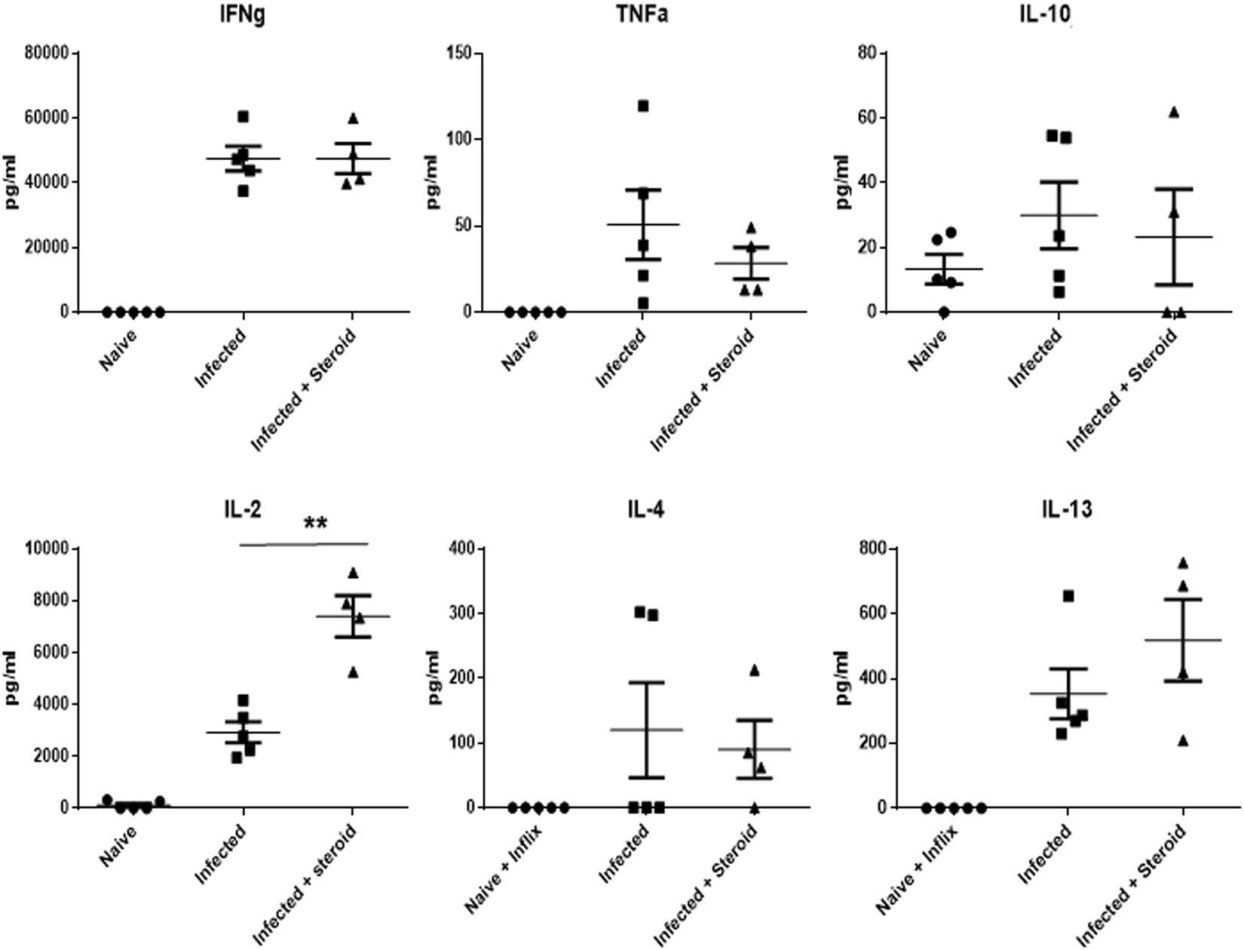
Hydrocortisone treatment significantly upregulated antigen specific IL2 production. Mesenteric lymph node (MLN) cytokine profiling in response to *T*. *muris* infection and steroid treatment. MLN cells were stimulated *in vitro* with *T*. *muris* specific E/S-antigen for 24 hours. The supernatant was analysed by cytokine bead array. IL-2 (p = 0.003) was significantly elevated after steroid therapy. Data are expressed for individual mice, and as mean value ± SD (n = 5 Naïve and Infected groups, n = 4 Infected + Steroid group).

MLN cells were re-stimulated *in vitro* with *T*. *muris*-specific Excretory/Secretory (ES) antigen day 45 post-infection. Cytokine production per 1 × 10^6^ cells is shown in Fig. 3. Of the cytokines assayed, only IL2 was significantly upregulated in the steroid treated group (p < 0.01). Interestingly, MLN cell number was dramatically reduced in steroid treated mice, therefore net cytokine production was considered. Of the cytokines measured, IFNι and IL13 production reduced proportionately with cell number although this was not significant (data not shown).

### Hydrocortisone significantly affected T cell associated colonic transcriptional activity

**Figure 4:**
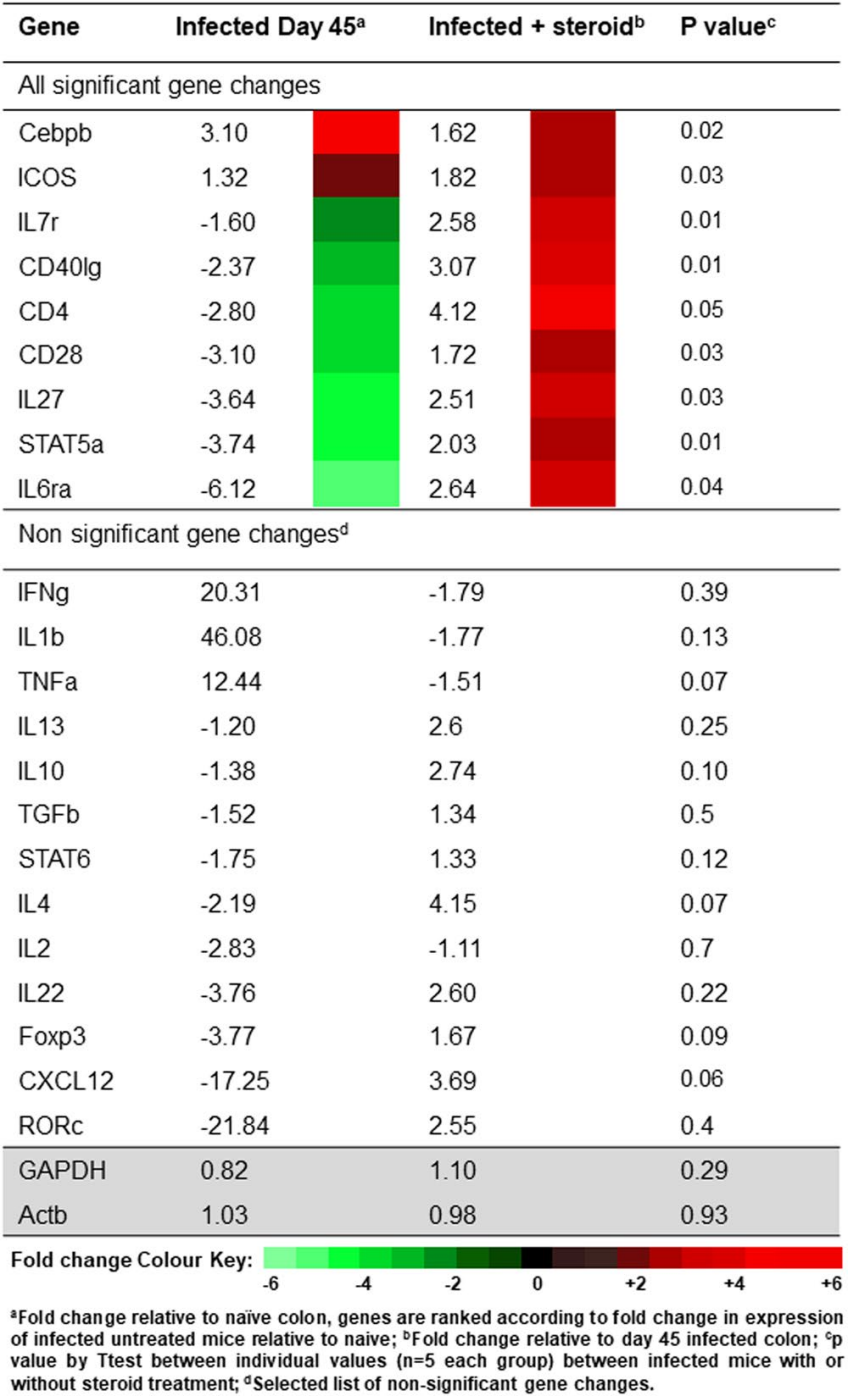
Altered regulation of key genes between *T*. *muris*-infected and steroid treated groups. Fold change in the colonic expression of adaptive immune response genes between day 45 steroid treated versus untreated *T*. *muris-*infected mice, and *T*. *muris* infected mice after hydrocortisone treatment. Fold changes are ranked according to fold change in expression of infected untreated mice relative to naïve.

No statistical difference between array genes was demonstrated between day 35 and day 45 infected AKR/J, except for two genes: *Stat3* (+1.55 fold change at day 45; p = 0.006) and *Stat5a* (+1.52 fold change at day 45; p = 0.043) (Fig. S1). As previously shown^11–13^, colonic gene expression differed significantly between naïve mice and those with chronic *T*. *muris* induced inflammation, demonstrating a dominant T_H_1 immune response (e.g. IFNι 20 fold increase, TNFα 12 fold increase compared to naive, Fig. 4).

The altered regulation of key genes between *T*. *muris*-infected and steroid treated groups are also shown in Fig. 4. Fold change after steroid treatment is represented relative to the infected cohort. Steroid treatment demonstrated modest reductions in T_H_1 (−1.79 fold reduction in IFNg gene expression compared to infected mice) although this was not significant. However, all genes with a significant fold change after steroid treatment (10% of those assayed, all upregulated) were associated with T cell homeostasis and maintenance (upper section, Fig. 4). For instance IL7r and downstream signaling molecule STAT5a were both significantly upregulated (+2.58 (p = 0.01) and +2.03 (p = 0.01) respectively). Similarly ICOS was also upregulated (+1.82 fold change, p = 0.03), known to promote the induction and expansion of CD4^hi^ iTreg (CD4 + 4.12 fold change, p = 0.05)^14^. IL-27 (+2.51 fold change, p = 0.03) has been shown to reduce colitis in mice by increasing the production of IL-10^15^ and Cebpb (C/EPBβ), a gene induced by retinoic acid, can enhance and stabilize Foxp3 expression in Treg cells during inflammation^16,17^ (+1.62 fold change, p = 0.02). Interestingly, although steroid treatment has been shown to inhibit Th17 cell development, we did not see any significant changes in IL-17 family members (0.14 < p < 0.45). Similarly IL6 was also unchanged (p = 0.33).

### Colonic Foxp3+ cell number were not affected by steroid intervention

**Figure 5:**
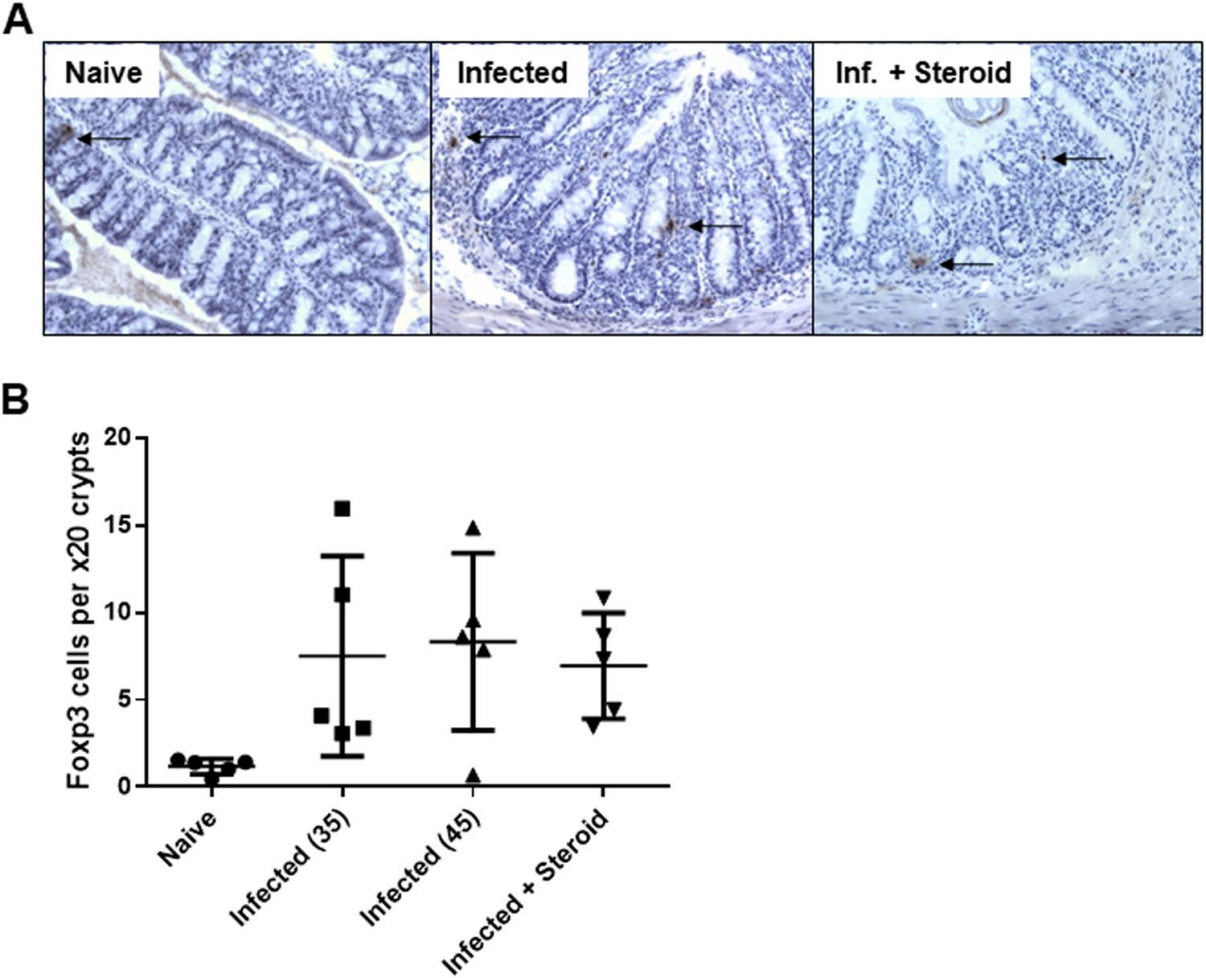
Hydrocortisone intervention does not increase Foxp3^+^ cells within the colonic tissue of *T*. *muris* infected mice. Immunohistochemistry staining for intracellular Foxp3 was performed on colonic cryosections (n = 5 per group). (**A**) Representative micrographs are shown (x200 magnification). Positive cells stained brown (arrows). (**B**) An increase in Foxp3^+^ cells was detected in all infected groups (data represented as the mean ± SEM). No significant difference in Foxp3^+^ cell numbers was found after steroid treatment (p = 0.61). Data representative of one experiment.

Given the elevated expression of factors associated with T cell regulation after steroid treatment, Foxp3+ cells were quantified by immunohistochemistry. Colonic foxp3^**+**^ cells increased significantly within all infected groups compared to naïve mice (p = 0.003) (Fig. 5). However, no statistical difference was identified between treated or untreated infected groups at day 45 post-infection after 10 days treatment with hydrocortisone.

## Discussion

We have previously shown that *T*. *muris*-induced T_H_1 driven colitis is a highly predictable model to study chronic colonic inflammation^11^. Here we chose to investigate hydrocortisone treatment, a key therapeutic agent for chronic inflammatory disease to study downstream immunological effects. The advantage of this model in the context of therapy is that treatment did not influence infection outcome *per se*, as all experimental groups harbored the same infection or worm burden.

Despite the identical and persistent antigenic insult, body weight stabilization and the partial restoration of bowel length was observed ten days following steroid treatment. The histological mild-to-moderate transmural colitis showed significant improvement with steroid therapy, demonstrating the initiation of immune suppression as expected. Although IFNι and IL13 cell cytokine secretion remained unchanged after re-stimulation of whole lymph nodes, IL2 increased significantly in the steroid treated group suggesting an environment conducive to T cell survival and differentiation., and one that has been shown to protect Th1 and Treg cells from glucocorticoid induced apoptosis^18^. Importantly, no significant changes were seen in transcription of IL-17 family members, Supporting Data demonstrating IL-17 and IL-17F in particular are resistant to glucocorticoid suppression^19^.

Local changes in gene transcription were highly indicative of enhanced T cell function. Obvious candidates for influencing T cell responses such as CD4, CD28, IL7r, STAT5a and CD40lg were some of the small number of genes (9/89 assayed) that were significantly upregulated. Upregulation of Cebpb or CCAAT/enhancer-binding protein beta (C/EBPβ) was particularly interesting. C/EBPβ has been known for some time to be upregulated in response to cortisol^20^ and to cooperate with STAT5 in transactivation of various genes^21–23^. C/EBPβ is known to be involved in the differentiation and proliferation of many cell types including dendritic cells and macrophages^24–27^. Dysregulation is frequently associated with increased inflammatory responses^28,29^, and many studies have demonstrated that C/EBPβ is essential in maintaining homeostasis of the T cell and haemopoetic compartments. Recently, Collins *et al*.^16^ have shown that C/EBPβ plays an essential role in Foxp3+ T cell function; Treg from C/EBPβ deficient mice had reduced IL-10 secretion and reduced functional ability to suppress T cell proliferation *in vitro*. Furthermore, T cells from C/EBPβ deficient mice induced more severe colitis *in vivo*.

It is worth noting that given the significant upregulation of several genes associated with induction of Treg cells, significant changes in downstream cytokines and associated growth factors might have been expected. It has been shown previously that glucocorticoid treatment of mice can upregulate regulatory genes such as TGFα and foxp3^30^, although a higher equivalent dose was used (20 mg/kg versus 5 mg/kg used in our study). However after 10 days of a pediatric equivalent dosage of hydrocortisone, significant changes in IFNι, TNFα, TGFβ, IL10 and IL22 genes were not seen. Despite trends towards up or downregulation of regulatory/inflammatory genes respectively, these did not reach significance despite observing a significant reduction in pathology. Of note, Colonic Foxp3+ cell number were not affected by steroid intervention and recent findings have confirmed this observation whereas the total number of foxp3+ Tregs would not affect the overall tolerogenic role of Tregs in the system. This could be a redistribution of sorts, similar to a mobilization of foxp3+ cells during the infection without affecting the overall functionality^30^.

Taken together, these data suggest that in the early stages of low dose hydrocortisone treatment, immune suppression is enabled via upregulation of several T cell associated genes enhancing regulatory function. Interestingly, these data also demonstrate that the significant transcriptional changes are upstream of genes known to be affected by longer term or higher dose hydrocortisone therapy, such as IFNι, IL-4, IL-13, IL-10^31^ and TNFα. These observations corroborate previous studies which have shown that glucocorticoid treatment induces immune suppression and can restore Treg function^32–34^ although the associated transcriptional profile has not been described to date. We suggest that enhanced expression of C/EBP-beta during hydrocortisone therapy, indicates T cell responsiveness, and may discriminate between early non-responders and those who will benefit from longer term glucocorticoid treatment^35^. Furthermore, this early marker of regulation may help determine a minimum therapeutic dose for individual patients.

## Methods and Materials

### Ethics

All experiments and procedures at the University of Manchester were sanctioned by The University of Manchester ethical review committee (UREC), and by the Home Office Animal Procedures Inspectorate. All experiments were carried out under the Home Office Scientific Procedures Act 1986 (revised January 2013).

### Animals

6 to 8 week old male AKR/J mice (Harlan Olac, Ltd., UK) were housed under specific pathogen-free conditions with free access to food and water. Mice were housed at the University of Manchester in a 24 °C temperature-controlled environment with a 12-hour light/dark cycle and free access to food and water.

### Induction of experimental colitis

*Trichuris muris* parasites were harvested and their ova extracted and maintained as previously described^36^. Infected AKR/J mice received 300 embryonated *T*. *muris* eggs in distilled water by oral gavage. Colitis was established by day 35 post infection.

### Experimental protocol and treatment

All experiments were concluded at day 35 or 45 post-infection. Hydrocortisone (Solu-cortef®, Pharmacia Ltd., UK) was given intra-peritoneally (100 ul i.p.) on alternate days from day 35 post infection. The dosage of steroid administered was equivalent to recommendations for active pediatric Crohn’s disease (2 mg/kg, QDS).

### Phenotyping

Stool appearance, body weight and well-being were monitored throughout the experiment. Phenotypic analysis was carried out at either day 35 or 45 post-infection for all mice as detailed in the text. Serum samples, mesenteric lymph node (MLN), and intestines were taken at autopsy. The colon was removed and measured from ileo-caecal valve to rectal margin. 0.5 cm of whole colonic tissue immediately distal to the ileo-caecal valve was isolated for RNA extraction (AllProtect®, Qiagen, UK). For histology, 0.5 cm of whole colonic segments from the proximal ascending colon were taken and fixed in neutral buffered formalin. A further 0.5 cm of ascending colonic tissue was snap frozen for immunohistochemistry. The remaining large bowel tissue was assessed for worm burden. Adults worms were pulled carefully out of caecum and proximal colon as described previously^37^.

### Histological evaluation

Samples were fixed in neutral-buffered formalin, processed in paraffin wax and 5 μm sections were stained with H&E. Slides were randomised and blinded. The assessment of mucosal architecture, cellular infiltration, muscle thickening, the presence or absence of crypt abscesses, goblet cell depletion, and ulceration was blindly interpreted by a clinical gastrointestinal histopathologist (Supplementary Table S1). Colonic crypt length and muscle thickness (μm) were measured using Image J software (http://rsbweb.nih.gov/ij) following the capture of images using SPOT™ Imaging Solutions camera and Advanced SPOT software (Diagnostic Instruments Inc., USA).

### Cytokine analysis

At autopsy, MLN were immediately transferred to wash buffer (RPMI 1640 medium supplemented with 2% FCS and 5 ml penicillin/streptomycin 100× (100 U/ml penicillin, 100 μg/ml streptomycin)) and cells isolated under sterile conditions on the day of harvest. Cells were washed twice, before re-suspension in complete media (RPMI 1640 medium supplemented with: 10% FCS, 5 ml (2 mM) L-glutamine, and 5 ml penicillin/streptomycin 100×). 1 × 107 cells/ml were stimulated, in triplicate, with either 50 μg/ml T. muris parasite excretory-secretory (ES) antigen^36,37^ or2.5 μg/ml of Con A (Sigma-Aldrich) and cultured (37 °C, 5% CO2, 24 hrs). Supernatants collected and stored at −20 °C until analysed. Serum was stored at −20 °C until analysed. MLN and serum cytokine proteins were measured using the FlowCytomix™ Mouse Kits Th1/Th2/Th17/Th22 13 plex Kit (eBiosciences, UK), and additional IL-12(p70) FlowCytomix™ Mouse Simplex Kit (eBiosciences, UK). FlowCytomix™ Pro 2.4 software (eBiosciences, UK) was used for data analysis.

### Colonic tissue RNA isolation and cDNA synthesis

After 24 hours storage in 1.0 ml AllProtect® solution (Qiagen, UK) at 4 °C, colonic tissue was carefully cleared of any luminal content, and tissue stored at −20 °C until processed. Individual colonic tissue samples (20 mg) were homogenized using Lysing Matrix D tubes® (MP Biomedicals) with a FastPrep®-24 homogenizer (MP Biomedicals) (4.0 M/sec). RNA was extracted from the supernatant using AllPrep® DNA/RNA/protein column kits according to manufacturer’s instructions (Qiagen, UK). Following an initial genomic DNA elimination step (SABiosciences), cDNA was synthesized using 1 μg of total RNA and a RT^2^ First Strand Kit (SABiosciences, USA) according to manufacturer’s instructions. cDNA was stored at −20 °C.

### QPCR and data analysis

For gene expression analysis, the Mouse T_H_17 for Autoimmunity and Inflammation (PAMM-073A) RT^2^ Profiler™ PCR Array (SABiosciences, USA) was employed throughout, according to manufacturer’s instructions. A complete table of 89 analysed genes is available at http://www.SABiosciences.com. A five-step cycling programme was used for Real Time and Melt Curve calculations as recommended (SABiosciences), and quantitatively analysed using a Bio-Rad MyIQ™ PCR detection system (Bio-Rad IQ5 optical system software, version 2; Bio-Rad Laboratories Inc.,©). ∆∆C_t_ PCR array data analysis, that incorporated genomic DNA contamination and Reverse Transcription impurities, utilized the SABiosciences Web Portal (http://www.SABiosciences.com/pcrarraydataanalysis.php) to analyse relative gene expression data between study samples and groups. To define the impact of therapy on chronic *T*. *muris*-induced colitis, fold change in colonic gene expression for the steroid treated group was analysed relative to day 45 post-infection untreated controls (Fig. 4).

### Immunohistochemistry

For the processing of frozen sections, 5 μm sections of ascending colon were fixed in 4% paraformaldehyde (4 °C, 10 minutes), and endogenous peroxidase activity was quenched (0.064 mg/ml sodium azide, 1.54 U/ml glucose oxidase and 1.8 mg/ml D-glucose, Sigma-Aldrich) at 37 °C for 20 minutes.

For Foxp3 detection, all washing was performed in 0.1% saponin/PBS. Non-specific binding sites were blocked with 10% goat serum (Sigma-Aldrich; 1 hour, RT). Endogenous avidin and biotin binding-sites were then blocked (Vector Laboratories, Peterborough, UK). Samples were incubated with purified rat anti-mouse FoxP3 (FJK-16s, eBiosciences, San Diego CA.; 2 μg/ml, 1 hour, RT), followed by a biotinylated goat anti-rat IgG secondary antibody (Santa Cruz Biotechnology, Inc., CA.; 2 μg/ml, 1 hour, RT.). An avidin-biotin horseradish peroxide macromolecular enzyme complex kit (Vectastain® ABC kit, Vector Laboratories; 30 minutes, RT.) was used for detection, followed by the addition of 3,3′-diaminobenzidine (DAB) peroxidase substrate (Vector Laboratories). Sections were counter-stained in Harris’s haematoxylin.

The number of positively stained cells was assessed at x400 magnification. After randomization and blinding, counting was repeated in triplicate for each section and data represented as mean per sample.

### Statistical analysis

Statistical analysis was performed using GraphPad PRISM® Version 5.00 (GraphPad Software, Inc.), with ANOVA and unpaired student *t*-test post-hoc analysis. Data are expressed as mean ± standard error of the mean.

## Supplementary information

Supplementary figures (T1, T2 and Sup figure 1)
