# Author Correction: Early induction of C/EBPβ expression as a potential marker of steroid responsive colitis

**DOI:** 10.1038/s41598-021-95179-0

**Published:** 2021-08-03

**Authors:** Mushref Bakri Assas, Scott Levison, Joanne L. Pennock

**Affiliations:** 1 Faculty of Applied Medical Sciences, Department of Medical Laboratory Technology, King AbdulAziz University, Jeddah, Saudi Arabia; 2 School of Biological Sciences, Faculty of Biology Medicine and Health, University of Manchester, Manchester, UK; 3 Manchester Royal Infirmary, Manchester University NHS Foundation Trust, Manchester, UK; 4 Lydia Becker Institute of Immunology and Inflammation, School of Biological Sciences, Faculty of Biology Medicine and Health, University of Manchester, Manchester, UK

Correction to: *Scientific Reports* 10.1038/s41598-019-48251-9, published online 11 September 2019

The original version of this Article contained an error in the spelling of the author Mushref Bakri Assas which was incorrectly given as Bakri M Assas. The original Article and accompanying Supplementary Information file have been corrected.

