# Supplementary material for: Early induction of C/EBPβ expression as a potential marker of steroid responsive colitis
Source: Sci Rep. 2019 Sep 11;9:13087. doi: 10.1038/s41598-019-48251-9 (PMC6739378; doi:10.1038/s41598-019-48251-9)

Early induction of C/EBPβ expression as a potential marker of steroid responsive colitis

Bakri M. Assas[1,2], Scott Levison[3], Joanne L. Pennock[4]

[1] Faculty of Applied Medical Sciences, Department of Medical laboratory technology, King AbdulAziz University, Jeddah, Saudi Arabia. massas@kau.edu.sa

[2] School of Biological Sciences, Faculty of Biology Medicine and Health, University of Manchester, Manchester, UK Mushref.Assas@manchester.ac.uk

[3] Manchester Royal Infirmary, Manchester University NHS Foundation Trust, Manchester, UK. Scott.levison@mft.nhs.uk

[4] Lydia Becker Institute of Immunology and Inflammation, School of Biological Sciences, Faculty of Biology Medicine and Health, University of Manchester, Manchester, UK. Joanne.pennock@manchester.ac.uk

Table S1. Criteria for microscopic scoring of colonic inflammatory disease.

	Degree of cellular infiltration	Mucosal architectural distortion	Extent of muscle thickening	Ulceration/ haemorrhage	Absence/ presence of crypt abscesses	Absence/ presence of goblet cell depletion
Score						
	0 - 3	0 - 3	0 – 3	0 - 3	0 - 1	0 - 1

0 = absent, 1, 2, 3: mild, moderate, severe

0 = absent,; 1 = present

Maximum score = 14

Table S2. Microscopic scoring of colonic inflammatory disease for individual study groups.

	**Degree of cellular infiltration**	**Mucosal architectural distortion**	**Extent of muscle thickening**	**Ulceration/ haemorrhage**	**Absence / presence of crypt abscesses**	**Absence/ presence of goblet cell depletion**	**MEAN score Max = 14 (SEM)**
Naïve	0	0	0	0	0	0	0 (0)
Infected (35)	2	2 to 3	1 to 2	0	0	1	1 (0.48)
Infected (45)	2 to 3	2 to 3	1 to 2	0	0	1	7 (0.31)
Infected + Steroid	1 to 2	1	1	0	0	0 to 1	4 (0.7)

Supplementary Figure S1. The expression of all colonic genes detectable by PCR array analysis demonstrated little transcriptional variance between infected untreated cohorts under similar experimental conditioning. A comparison between infected (day 35) vs infected (day 45) data from the *Mouse T_H17 for Autoimmunity and Inflammation* plate-array (SABiosciences) revealed similar gene expression between matched groups (SABiosciences PCR data analysis web-tool). Black line indicates fold changes ((2 ^ (-DC$_t$)) of 1. Pink lines indicate a x2 fold-change in gene expression threshold.

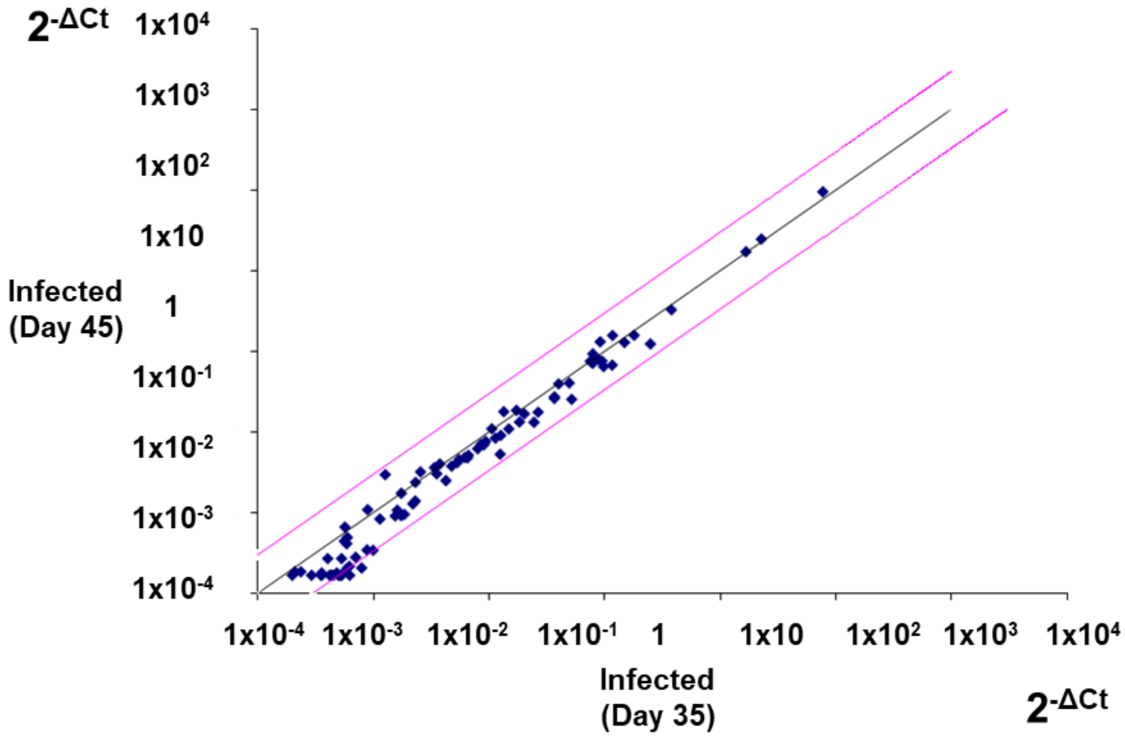

Supplement: Supplementary file 1 — Supplementary figures (T1, T2 and Sup figure 1) [file 41598_2019_48251_MOESM1_ESM.pdf]
